# Cysteine-Reactive Free ISG15 Generates IL-1β–Producing CD8α^+^ Dendritic Cells at the Site of Infection

**DOI:** 10.4049/jimmunol.1701322

**Published:** 2018-06-11

**Authors:** Anna Napolitano, Annemarthe G. van der Veen, Monique Bunyan, Annabel Borg, David Frith, Steven Howell, Svend Kjaer, Antje Beling, Ambrosius P. Snijders, Klaus-Peter Knobeloch, Eva-Maria Frickel

**Affiliations:** * Host-Toxoplasma Interaction Laboratory, The Francis Crick Institute, London NW1 1AT, United Kingdom;; † Immunobiology Laboratory, The Francis Crick Institute, London NW1 1AT, United Kingdom;; ‡ Structural Biology, The Francis Crick Institute, London NW1 1AT, United Kingdom;; § Protein Analysis and Proteomics Laboratory, The Francis Crick Institute, London NW1 1AT, United Kingdom;; ¶ Charité - Universitätsmedizin Berlin, corporate member of Freie Universität Berlin, Humboldt-Universität zu Berlin, and Berlin Institute of Health (BIH), Institute of Biochemistry, 10117 Berlin, Germany; and; ‖ Institute of Neuropathology, University of Freiburg, 79106 Freiburg, Germany

## Abstract

IFN-stimulated gene (ISG) 15 is a ubiquitin-like protein induced after type I IFN stimulation. There is a dearth of in vivo models to study free unconjugated ISG15 function. We found that free ISG15 enhances the production of IFN-γ and IL-1β during murine infection with *Toxoplasma gondii*. In our model, ISG15 is induced in a type I IFN–dependent fashion and released into the serum. Increased ISG15 levels are dependent on an actively invading and replicating parasite. Two cysteine residues in the hinge domain are necessary determinants for ISG15 to induce increased cytokine levels during infection. Increased ISG15 is concurrent with an influx of IL-1β–producing CD8α^+^ dendritic cells to the site of infection. In this article, we present *Toxoplasma* infection as a novel in vivo murine model to study the immunomodulatory properties of free ISG15 and uniquely link it to IL-1β production by CD8α^+^ dendritic cells driven by two cysteines in the hinge region of the protein.

## Introduction

The IFN-stimulated gene (ISG) 15 is a 15-kDa member of the ISG family that contains two ubiquitin-like domains connected by a proline peptide linker. Similar to ubiquitin, ISG15 can be conjugated to intracellular proteins through E1- (UBE1L), E2- (UbcH8), and E3-conjugating enzymes. ISGylation can modify protein function (1), and extensive studies have elucidated its role, mostly in the context of viral infections (1–4). Recently, the function of intracellular conjugated ISG15 has been extended to bacterial infections, in which it was found that *Listeria* infection induces ISGylation in nonphagocytic cells (5). Free ISG15 can also be released from the cell and is detectable in the serum (5–7). In vitro studies suggested a role for extracellular free ISG15 as a cytokine able to induce IFN-γ release from NK cells (4) and CD3^+^ lymphocytes (8, 9). However, only two studies addressed the in vivo function of free ISG15 (10, 11). In a neonatal model of infection with chikungunya virus, absence of free ISG15 increases proinflammatory cytokines in the serum, providing the first suggestion that free ISG15 contributes to the host response by regulating cytokine production during infection in the whole organism (10). Most strikingly, it was recently demonstrated that ISG15 is a potent proinflammatory IFN-γ–inducing cytokine in antimycobacterial immunity (11). Free extracellular ISG15 alone or in synergy with IL-12 induces IFN-γ secretion from granulocytes and NK cells in response to mycobacterial infection (11). The ISG15/IFN-γ circuit may therefore be an innate complement to the more adaptive IL-12/IFN-γ circuit (11). Both studies demonstrate that free ISG15 is critical for the survival of a chikungunya virus or *Mycobacterium tuberculosis* challenge in mice (10, 11).

*Toxoplasma gondii* is an obligate intracellular parasite that can infect virtually any nucleated cell. The parasite is never eliminated and establishes a chronic infection at immune-privileged sites, such as the brain and the heart (12). The acute infection is mostly controlled by IL-12 and IFN-γ, which orchestrate protective immunity in infected hosts (12), and their role has been extensively characterized (12, 13). Besides IFN-γ and IL-12, *T. gondii* infection triggers the release of a broad spectrum of molecules that can control the immune response to the parasite (12). In particular, recent work has drawn much attention to the role of the inflammasome and IL-1β in the control of *Toxoplasma* in mice, rats, and humans (14–17). In mice, *Toxoplasma* was shown to induce the IL-1β production via the inflammasome sensors NLRP1 and 3, ASC, and caspase 1/11 (15, 17).

We sought to delineate the molecular determinants of the in vivo activity of secreted ISG15 as proposed by Casanova and colleagues (11). To elucidate the mechanism of ISG15 action, we employed *Toxoplasma* infection, given its strong dependence on IL-12 and IFN-γ coupled with the parasite’s newly discovered ability to induce inflammasome activation. Moreover, we generated a double cysteine ISG15 mutant to reveal the molecular determinants essential for ISG15 in vivo function. In this study, we show that free extracellular ISG15 is produced and released in a type I IFN–dependent fashion and enhances IFN-γ and IL-1β levels during infection with live replicative *Toxoplasma* type II. As a prerequisite for its cytokine modulatory function, the cysteine residues C76 and C144 are required. We unequivocally show that ISG15 production during *Toxoplasma* infection results in the recruitment of CD8α^+^ dendritic cells (DCs) to the site of infection and that ISG15 exclusively stimulates IL-1β and not IL-12 production by CD8α^+^ DCs. These data demonstrate a novel role for ISG15 as a proinflammatory molecule that enhances IL-1β production within the context of an in vivo infection and highlight the essential requirement for its free cysteine residues to function in this capacity.

## Materials and Methods

### Mice

C57BL/6, ISG15^−/−^, and BATF3^−/−^ mice were housed and bred at the Francis Crick Institute, Mill Hill Laboratory, under specific pathogen-free conditions (9, 18). Ube1L-deficient mice were a kind donation from A. Beling (19, 20). Experiments were performed on 6- to 8-wk-old males. All procedures involving mice were approved by the local ethical committee of the Francis Crick Institute, Mill Hill Laboratory, and are part of a project license approved by the U.K. Home Office under the Animals (Scientific Procedures) Act of 1986.

### Parasite culture and infections

*Toxoplasma* avirulent type II Prugniaud strain (kind gift from M.-J. Gubbels and J. Saeij) (21, 22) was used for all the experiments. *T. gondii* cultures were maintained by serial passage on monolayers of human foreskin fibroblasts as described previously (23). Freshly egressed parasites were harvested from human foreskin fibroblast culture, filtered, counted, resuspended in PBS, and injected i.p. into mice. For in vivo imaging, *Toxoplasma* expressing GFP and firefly luciferase (21) was used. Mice were injected i.p. with 3 mg firefly d-luciferin (PerkinElmer), left for 10 min, and imaged with an IVIS Spectrum bioluminescence and fluorescence imaging system (Xenogen) under isoflurane anesthesia (Abbott Laboratories). For in vivo experiments, infected mice were treated with ISG15 (1 μg/mouse) or treated with PBS, buffer from the gel filtration, or nothing, with no difference observed.

### Reagents and Abs

Anti-ISG15 rabbit serum, a gift from K.-P. Knobeloch, was used as primary Ab for immunoblotting. Secondary Abs for immunoblotting were from KPL: goat anti-rabbit HRP (474-1506) and goat anti-mouse HRP (474-1806), used for the IgG H chain blot. Fluorescently labeled Abs for FACS against Ly6C (clone HK1.4), CD11b (clone M1/70), I-A/I-E (clone M5/114.15.2), CD11c (clone N418), Ly6G (clone 1A8), F4/80 (clone BM8), CD103 (clone 2E7), CD8α (clone 53-6.7), CD45R/B220 (clone RA3-6B2), CD49b (clone DX5), TCRβ (clone H57-597), and purified CD16/32 (anti Fc-γ receptor III/II, clone 93) were purchased from BioLegend. Anti-mouse IL-1β (clone NJTEN3), anti-mouse IL-12 (clone C15.6), and Rat IgG1 k isotype control (clone eBRG1) were purchased from eBioscience. Anti-FITC MACS beads (130-048-701) used for negative selection for the intracellular staining were from Miltenyi Biotec. ELISA Pierce TMB substrate kit (34021) was from Thermo Fisher Scientific. Dulbecco’s PBS was from VWR (21-031-CV).

### Immunoblotting

Immunoblotting was used to quantitatively detect specific protein bands after separation of proteins on precast 4–20%-gradient SDS-PAGE gel (4561095) from Bio-Rad. For reducing conditions with 2-ME, samples were treated with denaturing 8-M urea and boiled for 5′ at 95°C. Proteins were transferred onto nitrocellulose membrane (IB301002; Life Technologies) by dry blotting (iBlot; Life Technologies). Blots were blocked at retention time (RT) 1 h in a 5% nonfat dried milk/PBS solution (block buffer). The membrane was then incubated with primary Ab in 0.5% nonfat dried milk/PBS solution overnight at 4°C. The next day, blots were washed five times for 20 min in PBS with 0.1% Tween 20. Blots were incubated for 1 h with HRP-conjugated secondary Abs in block buffer with 0.1% Tween 20. Blots were washed five times for 20 min in PBS with 0.1% Tween 20. Finally, blots were developed with Immobilon Western Chemiluminescent HRP Substrate (Merck Millipore). Blots were exposed to CL-XPosure film (Thermo Fisher Scientific), and films were developed. Membrane was then treated with Western Blot Stripping Buffer (62299; Thermo Fisher Scientific) for 10 min RT and blocked again at RT 1 h in block buffer. After blocking, the membrane was incubated with the anti-mouse HRP secondary Ab for 1 h at RT and developed as before.

### Flow cytometry

Single-cell suspensions were prepared from spleen and lymph nodes (LNs) by mechanical disruption. Spleens were treated with the RBC Lysing buffer (RNBD7167; Sigma-Aldrich) for 5 min at room temperature to remove most of the blood cells. Peritoneal exudate cells were harvested by peritoneum lavage with 5 ml of PBS. After counting, cells were resuspended in PBS containing 1% BSA (PBA) and stained for 20 min at 4°C in an appropriate Ab mixture. Cells were washed twice with PBA before being analyzed by FACS.

For the intracellular staining, after harvesting, cells were stained with a mixture of FITC Abs and negatively selected with the anti-FITC beads according to the manufacturer’s instructions. Cells were then stained with the surface Abs in PBA, and BD Pharmingen Cytofix/Cytoperm kit (554714) was used for the intracellular staining according to the manufacturer’s instructions. Cells were run on a BD LSR II or BD LSR Fortessa X20 and analyzed using FlowJo software (Tree Star).

### Quantification of cytokines by ELISA

Mice were bled at different time points according to the experiment. Blood was left at RT for a minimum of 20 min, and serum was then isolated by centrifugation and stored at −80°C. Cytokines were analyzed by ELISA. Commercially available kits were used according to the manufacturer’s instructions to quantify the concentration of ISG15 (CY-8091; Caltag Medsystems), IL-1β (559603; BD Pharmingen), IFN-γ (555138; BD Pharmingen), and IL-12/IL-23 p40 (DY2398; R&D Systems).

### Real-time PCR

RNA was extracted using TRIzol reagent (10296010; Thermo Fisher Scientific) and quantified on the NanoDrop 3300 (Thermo Fisher Scientific). RNA was then reverse transcribed with the Superscript VILO cDNA Synthesis kit (11754050; Invitrogen) according to the manufacturer’s instructions. TaqMan Gene Expression Master Mix (4369016; Applied Biosystems) was used to perform real-time PCR in triplicate with 100 ng of cDNA per reaction using specific primers and GAPDH (Mm99999915_g1; Applied Biosystems) endogenous control on ABI Prism 7900 (Applied Biosystems), and results were analyzed with SDS 2.2.1 software. Relative quantitation of gene expression was determined using the comparative cycle threshold method (24). The specific primer used was from Applied Biosystems: Isg15 (Mm01705338_s1).

### Mass spectrometry analysis

Mouse serum was prepared under reducing or nonreducing conditions and subjected to SDS-PAGE. The SDS-PAGE loading buffer did not contain DTT to preserve oxidation states. Each of the two gel lanes was then cut into 20 roughly equally sized gel bands, and proteins were subjected to in-gel trypsin digestion. Peptides were extracted and aliquots were analyzed by liquid chromatography–mass spectrometry using a Q Exactive mass spectrometer operated in data-dependent acquisition mode. Next, two identified peptides corresponding to ISG15 were targeted for fragmentation in a parallel reaction monitoring (PRM) experiment targeting the masses 508.28 (IGVPAFQQR,2+) and 689.02 (GHSNIYEVFLTQTVDTLK,3+). The PRM scans were acquired at a resolution of 30,000 and were alternated with a full mass spectrometry scan (150–2000 m/z) at 70,000. Peak areas of precursor and fragment ions were calculated using Skyline software, and PRM spectra were searched using Mascot. Peaks that showed an RT shift of <1 min and received a Mascot score >10 were automatically accepted. Peaks with >1 min RT shift were rejected, and peaks with >1 min RT shift and a score <10 were manually curated.

### Purification of recombinant ISG15

Murine mature ISG15 fused to an HA tag, a TEV protease cleavage site, and a linker containing two glycine residues was cloned into the pTriEx6 expression plasmid. QuikChange mutagenesis (Stratagene) was used to introduce the C76S and C144S mutations. pTriEx6 containing the His-GST-3C-HA-TEV-GG-ISG15 gene was transfected into insect cells (Sf21 Berger) using the flashBAC system from Oxford Expression Technologies and FuGENE HD as transfection reagent. A total of 2.5 μl of flashBAC DNA, 5 μl of FuGENE HD, and 500 ng of pTriEx6 were used to transfect 4 × 10^5^ cells/ml in a six-well plate. Five hours posttransfection, 1 ml of Sf900 III containing Fungizone was added to the wells. The cells were incubated for 5 d at 27°C. The resulting 2 ml of P1 virus was used to infect 25 ml of Sf21 cells at 10^6^ cells/ml for 3 d at 27°C. The resulting P2 virus was titrated using quantitative PCR and amplified to 50 ml of P3 at a multiplicity of infection (MOI) of 1 (cells at 10^6^/ml). The P3 virus was used to infect a large scale of Sf21 cells, either at low density (cells at 10^6^/ml, MOI 1) or high density (cells at 6.10^6^/ml; MOI 3; glucose, lactalbumin, and Yeastolate added to the culture). The infected cells were pelleted and frozen at −80°C until purification. The intracellular ISG15 was purified from the Sf21 cell pellets using Glutathione Agarose (Cube Biotech) and Superdex 75 10/30 GL (GE Healthcare). Cells were lysed in 25 mM Tris (pH 7.5), 75 mM NaCl, 2.5% lycerol, 0.5% Triton, Benzonase Nuclease (Sigma-Aldrich), and Protease Inhibitor Tablets (Roche) using a sonication probe on ice. The lysate was then spun at 20,000 × *g* for 10 min at 4°C to pellet cell debris. The cleared lysate was incubated with Glutathione Agarose at 4°C for 2 h. The beads were washed three times with 25 mM Tris (pH 7.5), 75 mM NaCl, and 2.5% glycerol. The protein was cleaved using TEV protease overnight at 4°C. The cleaved protein was further purified by gel filtration in 25 mM Tris (pH 7.5), 75 mM NaCl, and 2.5% glycerol. The fractions containing pure ISG15 were pooled and concentrated. SDS-PAGE followed by Coomassie Brilliant Blue staining or immunoblotting with an anti-ISG15 Ab (clone A5, sc-166712; Santa Cruz Biotechnology) were used to verify purity of the recombinant product.

### Statistical analysis

All statistical significance analyses were performed using Prism software (GraphPad Software). Comparisons of data were performed using one-way or two-way ANOVA statistics with Tukey multiple comparisons test. Survival rates were compared by log-rank survival analysis of Kaplan–Meier curves.

## Results

### *T. gondii* infection is an in vivo model for studying cytokine production mediated by unconjugated ISG15

**FIGURE 1.**
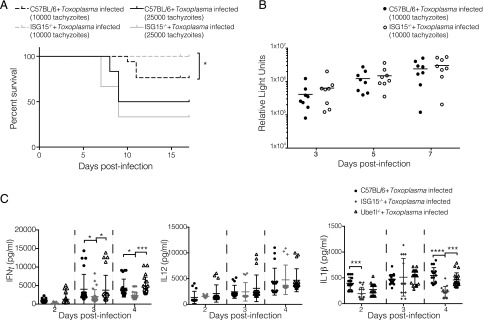
Extracellular free ISG15 modulates cytokine production during *Toxoplasma* infection. C57BL/6 and ISG15^−/−^ mice were infected with *Toxoplasma* type II tachyzoites i.p. (**A**) Survival of mice. Data combined from two and three independent experiments respectively, 14 mice per group for 1 × 10^4^ tachyzoites and 20 mice per group for 2.5 × 10^4^ tachyzoites. (**B**) Parasite load was measured by in vivo imaging of the firefly luciferase signal. (**C**) C57BL/6, UbE1L^−/−^, and ISG15^−/−^ mice were infected with 2.5 × 10^4^ *Toxoplasma* type II tachyzoites i.p. ELISAs for IFN-γ, IL-12, and IL-1β were performed on serum samples collected at different time points. Data combined from three independent experiments. Survival rates (A) were compared by log-rank survival analysis of Kaplan–Meier curves. Each individual point corresponds to a separate mouse (B and C). Two-way ANOVA statistical analysis with Tukey test of *Toxoplasma*-infected ISG15^−/−^, C57BL/6, and Ube1L^−/−^ mice (C). Only statistically significant relationships are shown. *p < 0.05, ***p < 0.0005, ****p < 0.00005.

The parasite *T. gondii*, akin to *M. tuberculosis*, is an obligate intracellular pathogen whose immune response is mainly characterized by a strong dependence on IL-12 and IFN-γ (12). Experimental infection of mice with *Toxoplasma* compared with *M. tuberculosis* is less time consuming and more tractable in terms of biological risk category. We therefore asked whether ISG15^−/−^ mice completely lacking ISG15 (12, 18) would withstand a *T. gondii* type II infection. We only observed a minor survival advantage of ISG15^−/−^ mice at a lower-dose *Toxoplasma* infection and no difference in survival or parasite load in the ISG15^−/−^ mice compared with C57BL/6 mice at a higher dose of infection (Fig. 1A, 1B). However, upon infection, levels of released IFN-γ and IL-1β cytokines were diminished in ISG15^−/−^ mice but not in control C57BL/6 mice or in UbE1L^−/−^ mice, which are devoid of intracellular conjugation of ISG15 through ISG15 E1 enzyme deficiency (12, 20) (Fig. 1C). This strongly suggests that the extracellular form of ISG15 decreases the production of IFN-γ and IL-1β during *Toxoplasma* infection, and ISGylation is not critical for this process. Importantly, this effect is not due to a general T cell and NK cell deficiency, as the ISG15^−/−^ mice have been shown to have the same cellular composition as C57BL/6 wild-type mice with regard to these immune components (18). Hence, we concluded that *Toxoplasma* infection represents a good in vivo model to study cytokine production mediated by unconjugated ISG15.

### Extracellular ISG15 is released during *T. gondii* infection

**FIGURE 2.**
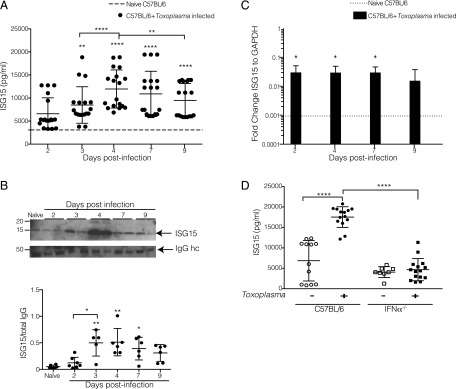
Extracellular free ISG15 levels increase upon *T. gondii* infection in a type I IFN–dependent way. (**A**–**C**) C57BL/6 mice were infected with 2.5 × 10^4^ *Toxoplasma* type II tachyzoites i.p., and serum samples were collected at different time points p.i. (A) Serum ISG15 levels during *Toxoplasma* infection were measured by ELISA. Data combined from three independent experiments. (B) ISG15 immunoblot on serum samples. Top panel, One representative blot of ISG15 and total IgG H chain used as loading control, two serum samples per infection time point. Bottom panel, Quantification of four independent immunoblot experiments. (C) Quantitative PCR for ISG15 transcripts on RNA extracted from peritoneal exudate cells from uninfected and at day 2, 4, 7, and 9 p.i. One of two independent experiments, six mice per group. (**D**) C57BL/6 and IFNαR^−/−^ mice were infected with 2.5 × 10^4^ *Toxoplasma* type II tachyzoites i.p., serum samples were collected at day 4 p.i., and ISG15 levels were measured by ELISA. Data combined from three independent experiments. Each individual point corresponds to a separate mouse (A, B, and D). Two-way ANOVA statistical analysis with Tukey test of *Toxoplasma*-infected versus naive C57BL/6 mice (A–C) and *Toxoplasma*-infected versus naive C57BL/6 and IFNαR^−/−^ mice (D). Only statistically significant relationships are shown. *p < 0.05, **p < 0.005, ****p < 0.00005.

To analyze the kinetics of ISG15 production in our model, we infected mice with *Toxoplasma* type II and monitored ISG15 release into the serum. As early as day 2 postinfection (p.i.), the amount of ISG15 in the serum was higher than in uninfected control mice (Fig. 2A), and its levels continued to rise and gain significance until day 4 p.i., after which the amount decreased (Fig. 2A). To ascertain the specificity of the commercial ELISA kit (19) we used to measure ISG15 levels, we demonstrated that no signal was observed in the serum of *Toxoplasma*-infected ISG15^−/−^ mice (Supplemental Fig. 1A). Likewise, recombinant ISG15 protein was detected in a dose-dependent manner (Supplemental Fig. 1B). We also analyzed the serum of *Toxoplasma*-infected mice by SDS-PAGE and immunoblotting and confirmed the release of ISG15 early upon infection (Fig. 2B top). The serum ISG15 protein band intensity of four experiments was quantified and normalized to IgG H chain (Fig. 2B, bottom). Therefore, through these results, we show that the free form of ISG15 is released in the serum of *Toxoplasma*-infected mice.

ISG15 belongs to the group of type I IFN–inducible genes and is highly induced after type I IFN–generating viral infections (3). The role of type I IFN during protozoan parasite infection is controversial and not yet fully understood (25). Recently, a transcriptomic survey of the host response to different *T. gondii* strains revealed that a subset of atypical strains induce a type I IFN response in macrophages and fibroblasts (19, 26). Other studies showed that *Toxoplasma* classical strains have the capacity to trigger a type I IFN response but have evolved strategies to limit the induction of type I IFN and the ability of type I IFN to activate STAT1-dependent transcription (10, 27, 28), highlighting new possible scenarios in the control of the parasitic infection. We analyzed the expression of *Isg15* in peritoneal exudate cells and we found that there is an induction of *Isg15* transcript at day 2 p.i., and this remained stable p.i. up until day 9, when *Isg15* was slightly reduced (Fig. 2C). To determine if the increase of ISG15 during the early phase of *Toxoplasma* infection is caused by type I IFN, we infected IFNαR^−/−^ mice with *Toxoplasma* type II and measured the level of ISG15 released in the serum 4 d p.i. As opposed to infection in C57BL/6 wild-type mice, infection of IFNαR^−/−^ mice did not result in increased ISG15 serum levels. Thus, we concluded that the induction of ISG15 mRNA is dependent on type I IFN signaling (Fig. 2D). It is of interest that the uninfected IFNαR^−/−^ mice already exhibit a lower level of ISG15 in the uninfected state as compared with C57BL/6 wild-type mice (Fig. 2D).

### Replicative *Toxoplasma* enhances the release of extracellular ISG15 to modulate serum cytokine levels

**FIGURE 3.**
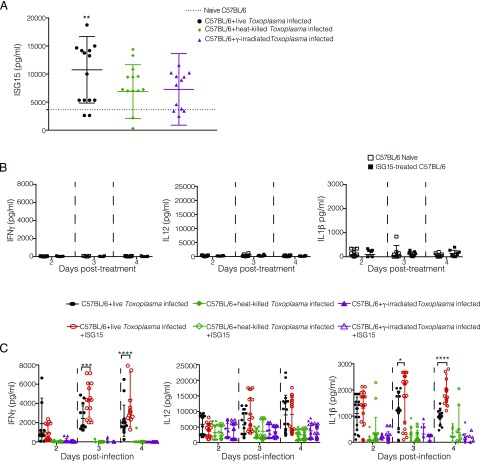
Cytokine modulation by extracellular ISG15 occurs during live *Toxoplasma* infection. (**A**) C57BL/6 mice were infected with live, heat-killed, or gamma-irradiated tachyzoites i.p., and serum ISG15 levels were assessed at day 4 p.i. Data combined from three independent experiments. (**B**) Uninfected naive C57BL/6 mice were left untreated or treated with recombinant ISG15 (1 μg/mouse) at day 0, 1, and 2. ELISA for IFN-γ, IL-12, and IL-1β was performed on serum samples collected at different time points posttreatment. Data combined from three independent experiments. (**C**) C57BL/6 mice were infected with 2.5 × 10^4^ *Toxoplasma* type II live parasites, heat-killed parasites, or gamma-irradiated parasites. For each condition, mice were either infected only or infected and treated with recombinant ISG15. ELISA for IFN-γ, IL-12, and IL-1β was performed on serum samples collected at different time points p.i. Data combined from three independent experiments. Each individual point corresponds to a separate mouse (A–C). Two-way ANOVA statistical analysis with Tukey test of *Toxoplasma*-infected versus naive C57BL/6 (A) and *Toxoplasma*-infected and *Toxoplasma*-infected plus recombinant ISG15–treated mice (C). Only statistically significant relationships are shown. *p < 0.05, **p < 0.005, ***p < 0.0005, ****p < 0.00005.

The release of ISG15 upon *Toxoplasma* infection might be an active process requiring the presence of a live, replicative parasite or be caused by recognition of specific parasite products such as pathogen-associated molecular patterns. To investigate whether ISG15 release depends on active host cell invasion and replication by the parasite, we infected mice with either live parasites, gamma-irradiated parasites that are able to invade but not replicate inside a cell, or heat-killed parasites that are phagocytosed by cells. When infection occurs with a live, active replicating parasite, we observed an increase in ISG15 levels as compared with gamma-irradiated or heat-killed parasites in the serum at day 4 p.i. (Fig. 3A). This suggests that enhanced ISG15 release during the early phase of infection is dependent on active invasion and replication of the parasite.

To dissect the role of free ISG15 during *Toxoplasma* infection, we next administered the protein in recombinant form in our model. Importantly for these experiments, treatment with ISG15 alone was not sufficient to cause an increase in cytokine secretion (Fig. 3B). This confirmed that the preparation of recombinant ISG15 was endotoxin free (Fig. 3B). Mice were infected with live, gamma-irradiated, or heat-killed *Toxoplasma* type II. In each case, we either only infected the mice or in addition treated them with 1 μg of recombinant murine ISG15 at days 0, 1, and 2 p.i. by i.p. injection. When mice were infected with live, actively replicating parasites, recombinant ISG15 injection increased IFN-γ and IL-1β levels compared with infected-only mice (Fig. 3C). The enhancement of IFN-γ and IL-1β production by recombinant ISG15 injection was not observed when the mice had been infected with gamma-irradiated or heat-killed parasites (Fig. 3C). This demonstrates that ISG15-dependent modulation of cytokine levels during the early phase of infection is dependent on active invasion and replication of the parasite and can be enhanced by administration of additional recombinant ISG15 into the serum.

### Cysteine residues in the ISG15 domain are required to modulate cytokine levels

**FIGURE 4.**
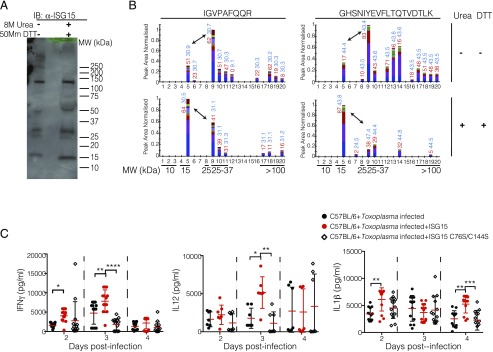
Cysteine residues of free ISG15 are important to induce cytokine release upon *T. gondii* parasite infection. (**A**) Serum from C57BL/6 mice infected with 2.5 × 10^4^ *Toxoplasma* type II tachyzoites at day 4 was run on SDS-PAGE under either nonreducing conditions or reducing conditions and followed by an anti-ISG15 immunoblot. Representative blot of three independent experiments. (**B**) Mass spectrometry quantification of ISG15 peptides IGVPAFQQR and GHSNIYEVFLTQTVDTLK. Peak areas indicating peptide abundance were extracted for precursor and fragment masses and are displayed as a cumulative bar. The mascot search engine score is displayed in red, and the RT is displayed in blue. Numbering on the *x*-axis starts at the bottom (low molecular weight [MW]) of the gel. Experiment performed once. Double arrow indicates comparison between putative monomeric (band 5) and dimeric (band 9) ISG15 species. (**C**) C57BL/6 mice were infected with 2.5 × 10^4^ *Toxoplasma* type II tachyzoites Prugniaud (Pru) and were either infected only or infected and treated with recombinant ISG15 or ISG15-C76S/C144S (1 μg/mouse) at days 0, 1, and 2 p.i. ELISA for IFN-γ, IL-12, and IL-1β was performed on serum samples collected at different time points p.i. Data combined from three independent experiments. Each individual point corresponds to a separate mouse (C). Two-way ANOVA statistical analysis with Tukey test of *Toxoplasma*-infected and *Toxoplasma*-infected plus recombinant ISG15– or ISG15 C76S/C144S–treated mice (C). Only statistically significant relationships are shown. *p < 0.05, **p < 0.005, ***p < 0.0005, ****p < 0.00005.

Immunoblot analysis of serum collected from wild-type mice infected with *Toxoplasma* type II at day 4 revealed the presence of different ISG15-specific bands with different m.w. (Fig. 4A). To test if these bands were cysteine-bound multimers of ISG15 or potential ISG15 conjugates, we performed an ISG15 immunoblot on serum treated in nonreducing and reducing conditions (Fig. 4A) and conducted protein mass spectrometry analysis on the serum samples (Fig. 4B, Supplemental Fig. 2A). We found that the quantity of monomeric ISG15 increased at the cost of higher m.w. ISG15-containing protein species when the serum was treated with reducing agent (Fig. 4B, band 5). Specifically, the band that contained the potential ISG15 dimer (Fig. 4B, band 9) and ISG15 multimers (Fig. 4B, bands 17–20) was dramatically less abundant after reduction of the serum. We thus concluded that *Toxoplasma*-infected serum contains cysteine-reactive ISG15 that is capable of forming multimers. This is consistent with a previous report identifying disulfide bonds between cysteines in the hinge region to be responsible for ISG15 dimerization or conjugation to other proteins (29) and with our analysis of the recombinant ISG15 protein used for the in vivo experiments (Supplemental Fig. 2B). To evaluate the role of the cysteine residues in the proinflammatory function of free ISG15, we infected mice with *Toxoplasma* and left them untreated or treated them with recombinant ISG15, or ISG15 in which the two cysteines in the hinge region were replaced by serines (ISG15-C76S/C144S). When injected into infected mice, only wild-type ISG15 had the capacity to increase the levels of cytokines in the serum (Fig. 4C). Thus, the presence of cysteine residues in the hinge region of the ISG15 protein are necessary to modulate cytokine release during the early phase of a live *Toxoplasma* infection.

### Free ISG15 increases IL-1β production by CD8α^+^ DCs at the site of infection

**FIGURE 5.**
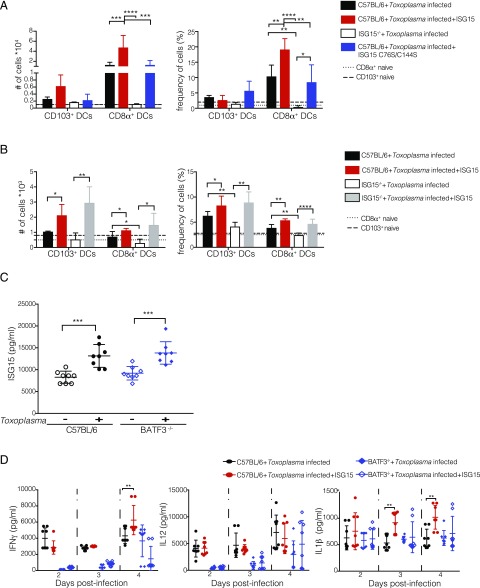
Free ISG15 increases IL-1β–producing CD8α^+^ DCs at the site of infection. (**A**) C57BL/6 and ISG15^−/−^ mice were infected with 2.5 × 10^4^ *Toxoplasma* tachyzoites i.p. C57BL/6 mice were either infected only or infected and treated with recombinant ISG15 or with ISG15-C76S/C144S mutant. At day 4 p.i., cells were isolated from the peritoneal exudate and FACS analysis was performed. CD8α^+^ and CD103^+^ DCs total cell numbers and cell frequency as percentage of total are shown. Three mice per group, one of four independent experiments. (**B**) C57BL/6 and ISG15^−/−^ mice were infected with 2.5 × 10^4^ *Toxoplasma* tachyzoites i.p. Mice were either infected only or infected and treated with recombinant ISG15. At day 4 p.i., cells were isolated from the spleen, and FACS analysis was performed. CD8α^+^ and CD103^+^ DC total cell number and cell frequency as percentage of total are shown. Three mice per group, one of two independent experiments. (**C**) C57BL/6 and BATF3^−/−^ mice were either uninfected or infected with 2.5 × 10^4^ *Toxoplasma* type II tachyzoites Prugniaud (Pru), and serum ISG15 levels were assessed at day 4 p.i. (**D**) C57BL/6 and BATF3^−/−^ mice were either infected only with 2.5 × 10^4^ *Toxoplasma* type II tachyzoites Pru or infected and treated with recombinant ISG15 (1 μg/mouse) at days 0, 1, and 2 p.i., and ELISA for IFN-γ, IL-12, and IL-1β was performed on serum samples collected at different time points. Data combined from two independent experiments. Each individual point corresponds to a separate mouse (C and D). One-way ANOVA statistical analysis with Tukey test of *Toxoplasma*-infected versus naive mice, ***p < 0.0005 (C). Two-way ANOVA statistical analysis with Tukey test of *Toxoplasma*-infected and *Toxoplasma*-infected plus recombinant ISG15–treated mice (A, B, and D). Only statistically significant relationships are shown. *p < 0.05, **p < 0.005, ***p < 0.0005, ****p < 0.00005.

IL1-β has only recently emerged as a contributor to the immune control of the parasite (2–4, 14, 17, 30), and therefore not much is known about its role and modulation during *Toxoplasma* infection. The finding that ISG15 modulates IL-1β release thus captured our interest. Consequently, we wanted to investigate which cells might release IL-1β in an ISG15-dependent manner during *Toxoplasma* infection. C57BL/6 mice were infected and left untreated or treated with recombinant ISG15. At day 4 p.i., we isolated cells from spleen, LNs, and peritoneal exudate and determined the identity of the innate cell compartment: neutrophils, macrophages, inflammatory monocytes, and DCs. In the spleen and LNs, we did not find any notable difference in any cell population in infected versus infected and ISG15-treated mice (Supplemental Fig. 3A). However, at the site of infection within the peritoneal exudate, we observed an increased number and frequency of DCs in ISG15-treated infected mice (Supplemental Fig. 3B). DCs play a critical role in the immune response to *Toxoplasma* infection (12, 13, 31). Among the different subsets of DCs, CD103^+^ and CD8α^+^ conventional DCs share a number of phenotypic characteristics, most likely derived from an immediate precursor that develops into either CD8α^+^ DCs or CD103^+^ DCs, depending on the tissue it seeds (12, 32). In the context of *Toxoplasma* infection, different studies have shown that CD8α^+^ DCs are a critical source of IL-12 during the acute phase of infection and relocate to the T cell area of the spleen to promote IFN-γ production by T cells (33, 34). Although CD8α^+^ DCs are mainly defined as lymphoid resident DCs, there are reports of migratory DCs expressing the CD8 marker present in lower frequency in different compartments (35). We therefore analyzed the DC compartment of the peritoneal exudate of *Toxoplasma*-infected ISG15^−/−^ and C57BL/6 mice upon infection, the latter left untreated or treated with recombinant ISG15 or with ISG15-C76S/C144S. At day 4 p.i., we observed a higher number and frequency of both CD103^+^ and CD8α^+^ conventional DCs in infected mice treated with ISG15, whereas a significantly lower number and frequency of these cells were observed in the ISG15^−/−^ infected mice. Mice treated with ISG15-C76S/C144S resembled untreated wild-type mice, confirming that cysteine residues of ISG15 are critical to facilitate CD8α^+^ DC recruitment to the site of infection (Fig. 5A, Supplemental Fig. 3C).

We noticed that *Toxoplasma*-infected ISG15^−/−^ mice presented with DC levels comparable to or lower than naive mice at the site of infection (Fig. 5A). To determine whether this is an intrinsic property of these knockout mice or if exogenously administered ISG15 could rescue the presence of DC, we infected B6 and ISG15^−/−^ mice and either left them untreated or treated them with recombinant ISG15. We analyzed the DC compartment in the spleen 4 d p.i. When ISG15^−/−^ mice are treated with recombinant ISG15 upon *Toxoplasma* infection, the levels of CD8α^+^ and CD103^+^ DCs are the same as those in C57BL/6 mice treated with recombinant ISG15 (Fig. 5A). These data confirm that free ISG15 regulates the number and frequency of DC subsets upon *Toxoplasma* infection.

To determine whether CD8α^+^ DCs are partially responsible for the increased IL-1β release in ISG15-treated mice, at day 4 p.i. we compared BATF3^−/−^ mice with C57BL/6 mice. It has been previously shown that deletion of the transcription factor Batf3 ablated the development of CD8α^+^ DCs (36). C57BL/6 and BATF3^−/−^ mice have similar serum ISG15 levels at day 4 post–*Toxoplasma* infection (Fig. 5C). When mice were infected with *Toxoplasma* and left untreated or were treated with recombinant ISG15, we found, as expected from the literature, that BATF3^−/−^ mice exhibit a greatly reduced IL-12 and IFN-γ production upon *Toxoplasma* infection (34), regardless of ISG15 administration (Fig. 5D). However, ISG15 injection did not enhance the levels of IL-1β present in the serum in BATF3^−/−^ mice as it does in C57BL/6 mice (Fig. 5D). Thus, we concluded that the presence of CD8α^+^ DCs is essential for ISG15 to be able to boost IL-1β levels during infection.

**FIGURE 6.**
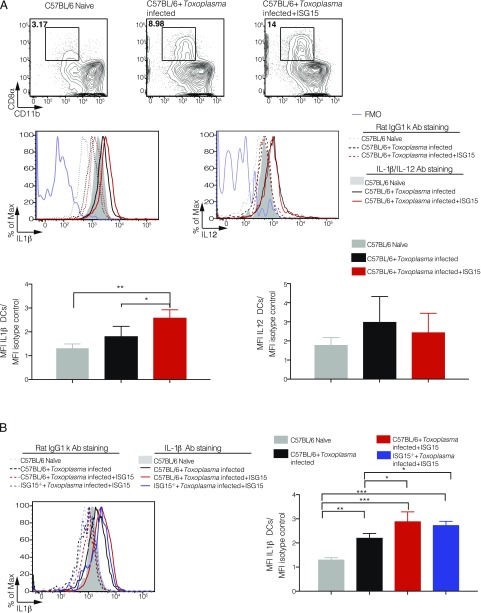
Free ISG15 exclusively recruits IL-1β–producing CD8α^+^ DCs. (**A**) C57BL/6 mice were either infected only or infected and treated with recombinant ISG15. At day 4 p.i., cells were isolated from the peritoneal exudate, and CD8α^+^ population was enriched via MACS cell sorting (negative purification). IL-1β and IL-12 intracellular staining was then performed on the enriched population. Top, Representative plot of one experiment. Bottom, MFI was calculated from IL-1β and IL-12 expression in CD8α^+^ DCs and normalized for the isotype control for each sample. Data combined from three independent experiments. (**B**) C57BL/6 mice were either infected only or infected and treated with recombinant ISG15, and ISG15^−/−^ mice were infected and treated with recombinant ISG15. Cells were isolated as in (A), and IL-1β intracellular staining was performed. Left, One representative plot. Right, MFI was calculated from IL-1β expression in CD8α^+^ DCs and normalized for the isotype control for each sample. Data combined from three independent experiments. Two-way ANOVA statistical analysis with Tukey test of *Toxoplasma*-infected and *Toxoplasma*-infected plus recombinant ISG15–treated mice. Only statistically significant relationships are shown. *p < 0.05, **p < 0.005, ***p < 0.0005.

To assess whether CD8α^+^ DCs directly and specifically produce IL-1β after ISG15 treatment at the site of *T. gondii* infection, we measured IL-1β and IL-12 by intracellular staining of this population. As a control for the specificity of the staining, we used both the fluorescence minus one setting and an isotype-matched control Ab (Fig. 6A). For each sample, we calculated the mean fluorescent intensity (MFI) of the cytokine expression in the CD8α^+^ cell population and normalized it to the isotype control of the same sample. Mice infected and treated with ISG15 exhibited increased IL-1β MFI expression in CD8α^+^ DCs, whereas the levels of IL-12 did not change between the infected and infected plus ISG15 samples (Fig. 6A). These results suggest that early in *T. gondii* infection, released ISG15 drives IL-1β production by CD8α^+^ DCs present at the site of the infection. Finally, as we have shown in Fig. 5B that recombinant ISG15 can rescue the levels of DCs in ISG15^−/−^ mice, we asked if the CD8α^+^ DC population in this setting is also capable of producing IL-1β. Indeed, we could demonstrate that exogenous ISG15 enhances IL-1β production directly from CD8α^+^ DCs in *Toxoplasma*-infected ISG15^−/−^ mice to the same levels as it does in *Toxoplasma*-infected C57BL/6 mice (Fig. 6B).

## Discussion

In this study, we demonstrate for the first time, to our knowledge, that *T. gondii* infection can be employed as an in vivo model to study the immunomodulatory properties of free extracellular ISG15. We show that free ISG15 is released into the serum and controls cytokine production during live *Toxoplasma* infection. We clearly demonstrate that the cysteine residues in the hinge region of ISG15 are mechanistically essential for this function to be intact. The presence of free ISG15 enhances the production of IL-1β by CD8α^+^ DCs present at the site of infection. ISG15 has been postulated to possess a cytokine-like role for more than 3 decades. Most studies have relied on in vitro stimulation, using recombinant ISG15, of PBLs, purified CD3^+^ T cells or CD56^+^ NK cells, or cell lines (8, 9). Only two in vivo studies have assessed the role of ISG15 as an immunomodulatory molecule (10, 37). In line with a previous report by the Casanova group (11), in this article, we show that ISG15 has an overall proinflammatory role and additionally extends its function from viral and bacterial to protozoan infections.

ISG15 is strongly induced upon type I IFN signaling activation (3). However, ISG15 can also be produced during bacterial listeriosis in a type I IFN–independent manner (5, 8, 9). The role of type I IFN during protozoan parasite infection is controversial and not yet fully understood (25). We demonstrate that the release of ISG15 is dependent on type I IFN. Additionally, the ISG15 response is not driven by a general damage-associated molecular pattern signal as its release requires the parasite to actively enter the cell, replicate, and initiate the inflammatory response. We can conclude extracellular ISG15 is produced as a consequence of an active infection to work as a cytokine-like molecule. Accordingly, although ISG15 alone is not sufficient to lower the parasite load, as demonstrated in Fig. 1A, it can increase IFN-γ and IL-1β levels. We additionally observed that the administration of recombinant ISG15 to *Toxoplasma*-infected mice often led to a bimodal distribution of increased IFN-γ and IL-1β when analyzing a population of mice. We hypothesize that this pattern may stem from the varying baseline free ISG15 levels we observe in naive mice. Regardless, by day 4 p.i., which coincides with the highest natural free ISG15 serum levels during *Toxoplasma* infection, we observe the least bimodal distribution of serum IFN-γ and IL-1β in response to exogenously administered ISG15.

ISG15 can be detected in both intracellular and extracellular forms (7–9). During the intracellular ISGylation process, ISG15 dimer formation, through conserved cysteine residues, reduces the amount of ISG15 that can be coupled with the target proteins, a process that can be prevented by nitrosylation of those cysteine residues on the ISG15 molecule (5, 29). Aside from the ISGylation process, it is unclear whether the quaternary structure of extracellular ISG15 is essential for its function. In this study, we show, in line with Okumura et al. (29), that *Toxoplasma*-infected serum contains cysteine-reactive ISG15 that is capable of forming dimers and higher order species. We also concluded that the cysteine residues present in the protein structure of ISG15 are important for the cytokine-like activity of free ISG15 in vivo during infection with *Toxoplasma*. The presence of these active cysteines is either required for de novo recognition by its putative receptor on target cells or for the downstream signaling response that ensues upon stimulation of its receptor. Interestingly, the recently identified ISG15 receptor LFA-1 on NK cells does not depend on the intact cysteine at this position in the hinge region of ISG15 to produce IFN-γ (38). We, in contrast, unequivocally demonstrated that free ISG15 not only possess a proinflammatory capacity during a protozoan infection but also employs its cysteines to exert this function. Although we cannot exclude another cell type or factor to be the sensor of free ISG15 based on our current results, it is tempting to speculate that CD8α^+^ DCs express a distinct ISG15 receptor and, in response to increasing ISG15 levels during *Toxoplasma* infection, migrate to the site of infection to produce IL-1β. It will be interesting to determine whether this observation is restricted to the infection with *Toxoplasma* or whether this is a general feature of free ISG15 function.

IL-1β is released upon a broad variety of stimuli, highlighting its essential role during immune response. Many cell types have been identified as IL-1β producers during infection (39, 40). We found that ISG15 levels correlate with an increase of IL-1β levels in the serum and with increased number and frequency of CD8α^+^ conventional DCs at the site of infection. We now show for the first time, to our knowledge, that along with IL-12, those cells can also release IL-1β during *Toxoplasma gondii* infection. In BATF3^−/−^ mice lacking CD8α^+^ DCs (36), ISG15 fails to induce an IL-1β increase during *Toxoplasma* infection, as seen in B6 mice, demonstrating that ISG15 induces IL-1β–producing CD8α^+^ DCs at the site of infection.

In our study, we employed the in vivo *Toxoplasma* infection model to define novel extracellular functions for ISG15. We show that *Toxoplasma* infection triggers the release of free ISG15. We found that free ISG15, via its cysteine residues, acts in vivo as an enhancer of IFN-γ and IL-1β released during the early phase of the immune response to *Toxoplasma* infection. The cysteine residues of free ISG15 are required to trigger an increase in CD8α^+^ DCs producing IL-1β at the site of the infection. Free ISG15 may therefore be a novel modulator of the immune response to an infection, and it remains to be investigated if other IL-1β activating infections can be influenced by ISG15. This may be a broader phenomenon and warrants further investigation.

## Supplementary Material

Data Supplement
