# Supplementary material for: Cysteine-Reactive Free ISG15 Generates IL-1β–Producing CD8α+ Dendritic Cells at the Site of Infection
Source: J Immunol. 2018 Jun 11;201(2):604–14. doi: 10.4049/jimmunol.1701322 (PMC6036233; doi:10.4049/jimmunol.1701322)
Supplement: Data Supplement [file JI_1701322.zip › JI_1701322_Supplemental_Figures_1.pdf]

Figure S1

A

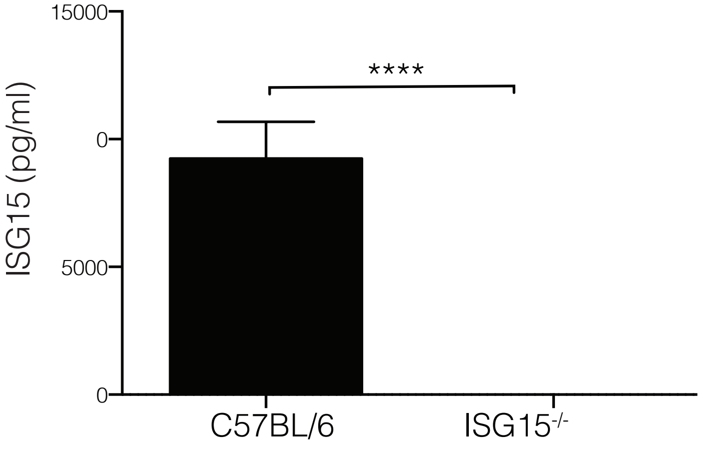

B

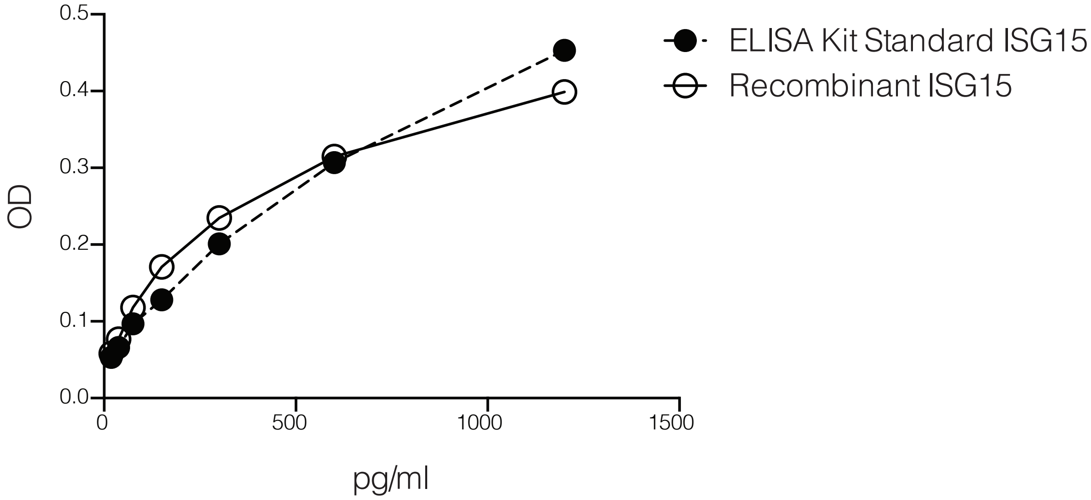

Figure S1: A) ISG15 ELISA on serum collected at day 4 p.i. from C57BL6 and ISG15$^{-/-}$ mice infected with 2.5×10^4 *Toxoplasma* type II tachyzoites i.p.. B) Recombinant ISG15 was used as a standard in comparison with the standard provided by the ELISA kit. Two way ANOVA statistical analyses with Tukey's test of *Toxoplasma* infected C57BL6 versus ISG15$^{-/-}$ mice. Only statistically significant relationships are shown; ****$p<0.00005$.

Figure S2

A

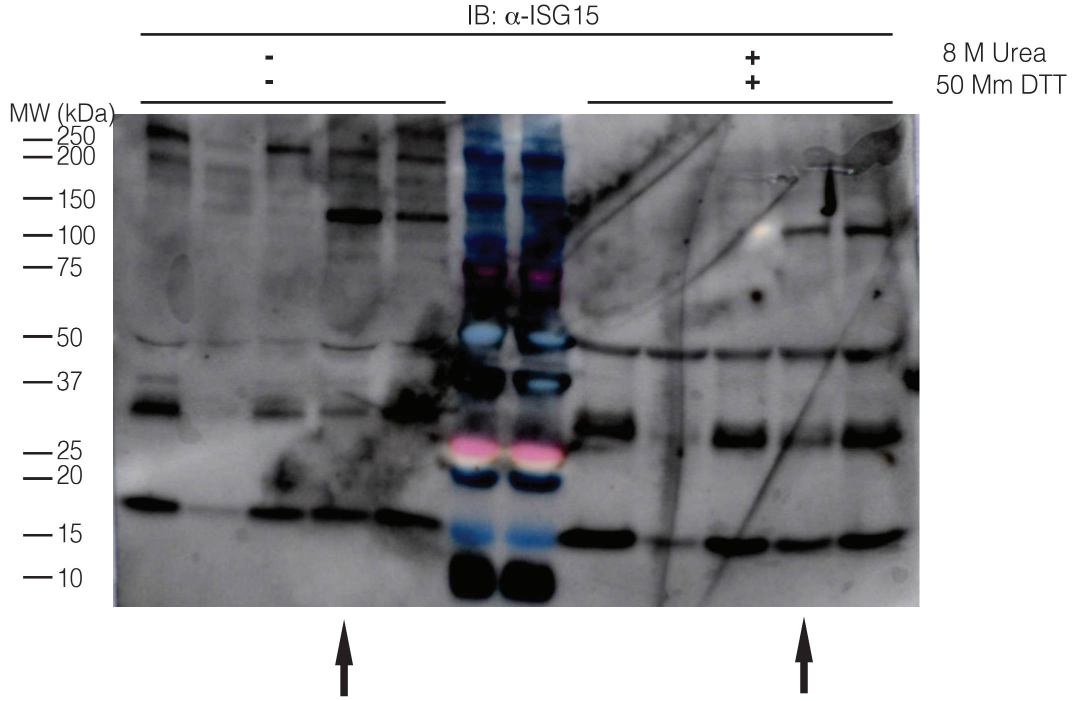

B

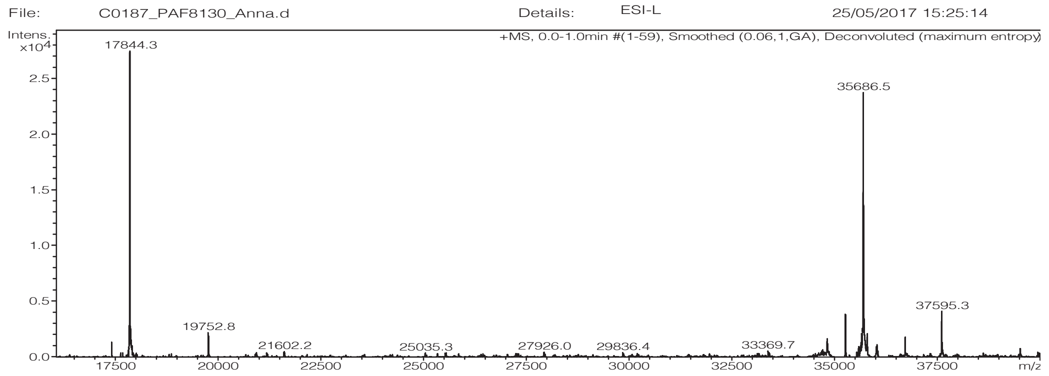

Figure S2: A) Serum from C57BL6 mice either infected with $2.5x10^4$ *Toxoplasma* type II tachyzoites at day 4 or uninfected was run on SDS-PAGE either with non-reducing conditions or with reducing conditions and followed by an anti-ISG15 immunoblot. Lane indicated with the arrow have been sliced and used for Mass Spectronomy. B) Recombinant ISG15 protein was analysed by Mass Spectronomy demonstrating the presence of a monomeric and single cysteine-reactive dimer species in the preparation.

Figure S3

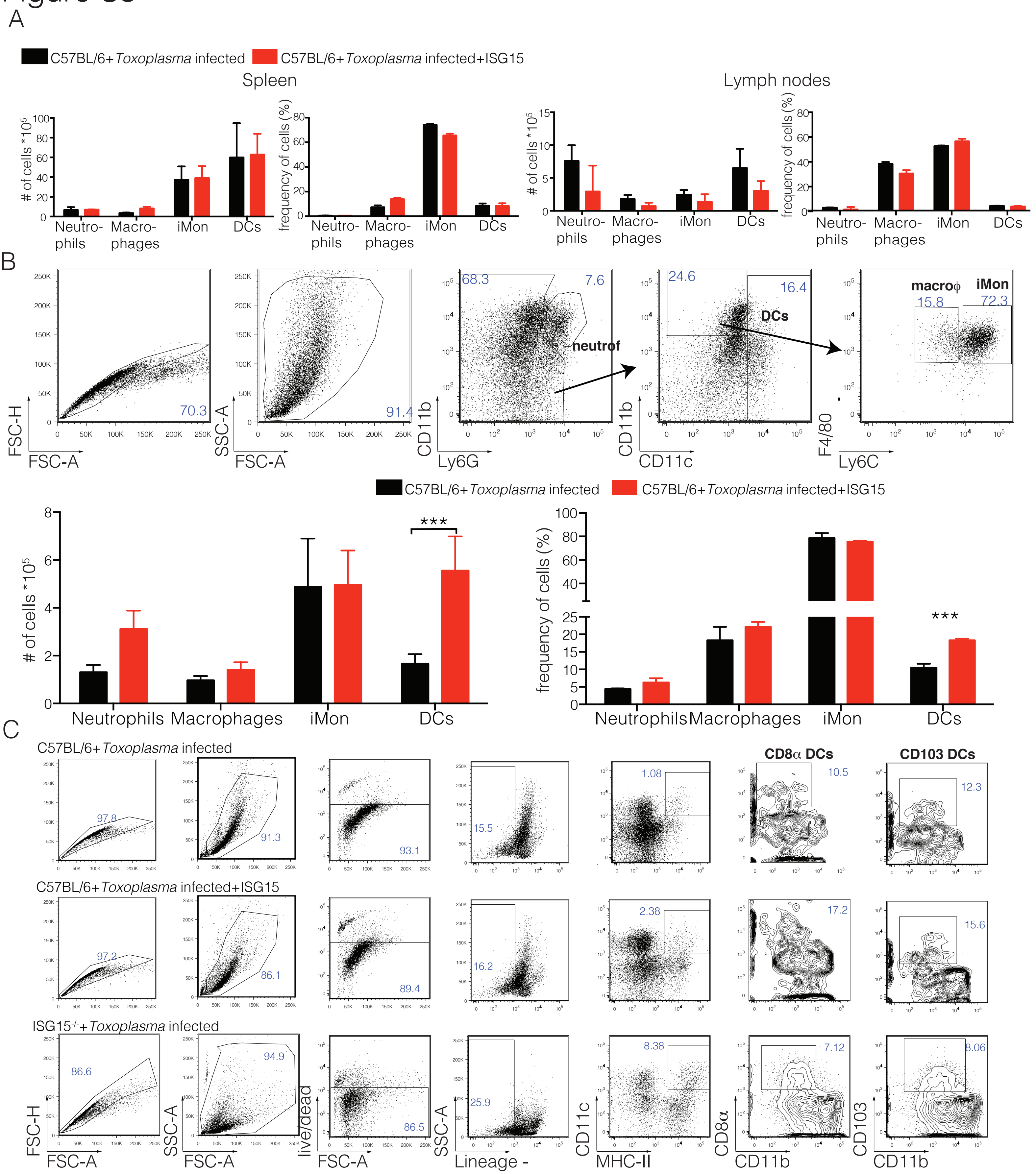

Figure S3: C57BL6 mice were either infected with 2.5×10^4 *Toxoplasma* type II tachyzoites i.p. and treated with recombinant ISG15 (1µg/mouse) at day 0, 1 and 2 post infection or infected only. A) Mice were sacrificed 4 days p.i. and cells were purified from draining lymph nodes (inguinal and brachial) and spleen. Number and frequency of the cell populations analysed is shown, 3 mice per group. One of three independent experiments. B) and C) Mice were sacrificed 4 days post infection and cells were purified from peritoneal exudate. B) Gating strategy (top) and quantification (bottom) for the staining used to analyse the innate cell populations, 3 mice per group. One of three independent experiments. C) Gating strategy for the staining used to analyse $CD8\alpha^+$ and $CD103^+$ DCs, 3 mice per group. One of three independent experiments. Two way ANOVA statistical analyses with Tukey's test of *Toxoplasma* infected C57BL6 versus infected and ISG15 treated mice. Only statistically significant relationships are shown; ***p<0.0005.
